# Skin absorption of felbinac solid nanoparticles in gel formulation containing l-menthol and carboxypolymethylene

**DOI:** 10.1186/s40780-023-00290-1

**Published:** 2023-06-06

**Authors:** Reita Kadowaki, Fumihiko Ogata, Aoi Fushiki, Saki Daimyo, Saori Deguchi, Hiroko Otake, Mayumi Nagata, Hiroshi Sasaki, Naohito Kawasaki, Noriaki Nagai

**Affiliations:** 1 Faculty of Pharmacy, Kindai University, 3-4-1 Kowakae, Higashi-Osaka, 577-8502 Osaka Japan; 2 Department of Ophthalmology, Dokkyo Medical University, 880 Kitakobayashi, Mibu, 321-0293 Tochigi Japan; 3 Department of Ophthalmology, Kanazawa Medical University, Ishikawa, 920-0293 Japan

**Keywords:** Felbinac, Skin formulation, Nanoparticle, *l*-menthol, Drug delivery

## Abstract

**Background:**

It is important to design an effective formulation to enhance the skin penetration, and nanotechnologies have been used in dermal and transdermal drug delivery. In this study, we prepared formulations (gels) containing *l*-menthol and felbinac (FEL) solid nanoparticles (FEL-NP gel) for topical application, and investigated the local and systemic absorption of the prepared FEL-NP gel.

**Methods:**

FEL solid nanoparticles were obtained by bead milling of FEL powder (microparticles), and a topical formulation (FEL-NP gel) consisting of 1.5% FEL solid nanoparticles), 2% carboxypolymethylene, 2% *l*-menthol, 0.5% methylcellulose, and 5% 2-hydroxypropyl-β-cyclodextrin (w/w %) were prepared.

**Results:**

The particle size of FEL nanoparticles was 20–200 nm. The released FEL concentration from FEL-NP gel was significantly higher than that from FEL gel without bead mill treatment (carboxypolymethylene gel in which FEL microparticles (MPs) instead of FEL nanoparticles were incorporated, FEL-MP gel), and FEL was released as nanoparticles from the gel. Moreover, both transdermal penetration and percutaneous absorption of FEL-NP gel were significantly increased compared with those of FEL-MP gel, and the area under the FEL concentration-time curve (*AUC*) of FEL-NP gels was 1.52- and 1.38-fold of commercially available FEL ointment and FEL-MP gel, respectively. In addition, after 24 h of treatment, the FEL content in rat skin treated with FEL-NP gels was 1.38- and 2.54-fold higher than that when treated with commercially available FEL ointment and FEL-MP gel, respectively. Moreover, the enhanced skin penetration of FEL-NP gels was significantly attenuated by inhibition of energy-dependent endocytosis, such as clathrin-mediated endocytosis.

**Conclusions:**

We successfully prepared a topically applied carboxypolymethylene gel containing FEL nanoparticles. In addition, we observed that the endocytosis pathway was mainly related to the high skin penetration of FEL nanoparticles, and FEL-NP gel application resulted in high local tissue concentration and systemic absorption of FEL. These findings provide useful information for the design of topically applied nanoformulations against inflammation by providing local and systemic effects.

**Supplementary Information:**

The online version contains supplementary material available at 10.1186/s40780-023-00290-1.

## Background

(2-(4-Phenylphenyl)acetic acid) (Felbinac; FEL), belonging to arylpropionic acid class, is a nonselective non-steroidal anti-inflammatory drug (NSAIDs) [1, 2] that attenuates swelling and pain from inflammation *via* prostaglandin secretion, particularly in the muscles and joints [3]. However, oral administration of FEL causes irritation in the gastrointestinal tract owing to the presence of free carboxylic groups; therefore, oral administration of FEL is contraindicated [4, 5]. Drug delivery through dermal and transdermal routes is preferred for FEL administration; topical administration of FEL has shown anti-analgesic and anti-inflammatory activities [6]. The skin formulation avoids degradation in the gastrointestinal tract, reduces side effects, provides pain-free and safe administration, reduces the frequency of application, eliminates hepatic first-pass metabolism, and ensures consistent serum levels of drugs [7]. In the clinical setting, transdermal formulations containing FEL, such as FEL ointment and patches, are available; however, transdermal absorption of the drug from FEL ointment is low. In addition, large application area of patches owing to low skin absorption is far beyond that of the desired size (less than 40 cm^2^) [8]. Low absorption of FEL through the skin results in low patient compliance [8].

Balanced lipophilicity (log P: 1–3) and low molecular weight (MW: <500 Da) are the required physicochemical properties of drugs to enable skin penetration; the primary skin absorption of drugs is led by passive diffusion [9, 10]. FEL has high oil solubility, low MW, good stability, and skin non-irritant properties; however, there are many other factors that influence dermal and transdermal drug delivery. Therefore, application of physical or chemical techniques is essential for enhancing drug permeation through the stratum corneum (SC) [9]. Several physical techniques such as needleless injection, iontophoresis, magnetophoresis, sonophoresis, microporation, and electroporation, and chemical techniques such as prodrugs, colloidal formulations, and permeation enhancers, have been reported [11, 12]. In addition, many studies have suggested that nanoparticles are useful for enhancing skin permeation through the SC [13, 14]. Kowalska and Kalinowska-Lis reported [15] that nanoparticles of size 40–800 nm adhere to the lipid layer of the SC, resulting in penetration of an enhanced number of drug molecules in the deeper skin layers. Moreover, absorption of drug nanoparticles may increase owing to the occlusion effect [15]. However, the precise mechanism of skin permeation of nanoparticles is not clear, although changes in drug particle size enhance the cellular uptake of nanoparticles [16]. Based on these findings, nanotechnologies, such as nanocrystals, nanoemulsions, dendrimers, and liposomes, have been used in dermal and transdermal drug delivery. We also reported that solid nanoparticles of raloxifene and indomethacin of size > 100 nm were obstructed in the SC, and only those of size < 100 nm could penetrate the skin tissue *via* the SC, resulting in the improved percutaneous absorption of raloxifene and indomethacin [17, 18]. These findings showed that the SC, which limits the skin permeability of nanoparticles, could be an obstacle for the design of skin formulations containing FEL nanoparticles. On the other hand, we found that *l*-menthol, an enhancer of drug skin permeation, altered the barrier properties of the SC, and solid nanoparticles of raloxifene and indomethacin of size 100–200 nm could penetrate through the SC when combined with *l*-menthol, resulting in the improved skin absorption of drug nanoparticles [17, 18]. In this study, we attempted to investigate whether formulations (gels) containing *l*-menthol and FEL solid nanoparticles (FEL-NP gel) for topical application enhance the local and systemic absorption of the FEL.

## Materials

### Animals

Seven-week-old male Wistar rats (weighing approximately 200 g) were provided by the Kiwa Laboratory Animals Co., Ltd. (Wakayama, Japan), and all animal experiments were performed according to the guidelines of Kindai University, the Japanese Pharmacological Society, and NIH. The experimental protocols were approved on April 1, 2019, by Kindai University under the project identification code KAPS-31-011, and the study was carried out in compliance with the ARRIVE guidelines and the AVMA euthanasia guidelines 2020. The rats were housed at 25 °C, and allowed free access to water and a standard CE-2 diet (Clea Japan Inc., Tokyo, Japan).

### Chemicals

We used commercially available chemicals of highest purity in this study. Briefly, FEL, *l*-menthol, cytochalasin D, and isoflurane were purchased from Wako Pure Chemical Industries, Ltd. (Osaka, Japan), and commercially available FEL stick ointment 3% (CA-FEL ointment) was obtained from Mikasa Seiyaku Co., Ltd. (Tokyo, Japan). Dynasore and rottlerin were provided by Nacalai Tesque (Kyoto, Japan), and carboxypolymethylene (Carbopol^®^ 934) was obtained from Serva (Heidelberg, Germany). Pentobarbital was obtained from Tokyo Chemical Industry Co., Ltd. (Tokyo, Japan). Methylcellulose (MC) was purchased from Shin-Etsu Chemical Co., Ltd. (Tokyo, Japan), and 2-hydroxypropyl-β-cyclodextrin (HPβCD) was provided by Nihon Shokuhin Kako Co., Ltd. (Tokyo, Japan). Membranes of pore size 20 μm for the drug release study were purchased from GVS Japan (Tokyo, Japan).

### Design of carboxypolymethylene gel-based l-menthol-containing FEL nanoparticles

Carboxypolymethylene gel incorporating FEL micro- or nanoparticles was prepared according to our previous reports [17–19]. Briefly, FEL was milled in an agate mortar for 1 h, which was subsequently transferred to 2 mL tube filled with 2 mm zirconia beads, and crushed (3,000 rpm, 30 s) at 4 °C using the bead mill method and Bead Smash 12 (Wakenyaku Co. Ltd, Kyoto, Japan). After that, FEL and MC were added to 5% HPβCD solution (in distilled water) and crushed using 0.1 mm zirconia beads and ShakeMaster^®^ NEO (Bio Medical Science, Tokyo, Japan). The bead mill treatments were performed for 180 min at 1,500 rpm, and the milled dispersion was ultrasonicated. These dispersions containing milled FEL were gelled by mixing carboxypolymethylene dissolved in distilled water with 2% *l*-menthol, and determined as the FEL-NP gel in this study. We prepared the gels incorporating FEL microparticles (FEL-MP gel) by dispersing FEL (original crystal, microparticles), MC, and HPβCD in distilled water, which was gelled by the mixing with carboxypolymethylene dissolved in distilled water with 2% *l*-menthol. The content of additives, such as *l*-menthol, were decided according to our previous study [17, 18], and the composition of the FEL-MP and FEL-NP gels was 1.5% FEL, 0.5% MC, 5% HPβCD, and 2% carboxypolymethylene [% (w/w) in distilled water].

### Particle characteristics of FEL gel

In this study, the particle size of FEL gel in dispersion containing FEL (0.3 g of FEL gel was diluted with 100 mL of distilled water and stirred for 10 min) was measured using SALD-7100 (Shimadzu Corporation) and NANOSIGHT Software NTA (Quantum Design Japan, Inc.). The measurement conditions for SALD-7100 were a maximum scattered light intensity in the range of 40–60% and a refractive index of 1.60 ± 0.10i. The measurement conditions for NANOSIGHT were a wavelength of 405 nm (Blue), time of 60 s, and viscosity of 1.27 mPa·s. In addition, dispersions containing FEL were set to a scanning probe microscope (SPM-9700; Shimadzu Corp., Kyoto, Japan) to obtain atomic force microscopy (AFM) images represented by the combination of the phase and height images of FEL.

### Measurement of FEL concentration

Fifty microliters of samples containing FEL were added to 100 µL of methanol and used to measure the FEL concentration using Shimadzu LC-20AT system equipped with a column oven CTO-20 A (Shimadzu Corp., Kyoto, Japan). The samples (10 µL) were injected using an auto injector SIL-20AC (Shimadzu Corp., Kyoto, Japan). The measurement conditions were as follows: column: Inertsil^®^ODS-3 (3 μm; GL Science Co. Inc., Tokyo, Japan); column temperature, 35 °C; flow rate, 0.25 mL/min; mobile phase, 0.3% phosphoric acid containing 1% sodium dodecyl sulfate-acetonitrile (70/30 v/v); detection wavelength, 254 nm.

### Viscosity of FEL gel

The viscosity of FEL gel was measured using Brookfield digital viscometer with a CPZ-52Z plate (Brookfield Engineering Laboratories, Inc., Middleboro, MA, USA). The measurement conditions were as follows: rotational speed, 60 rpm; measurement time, 3.5 min; and temperature, 20 °C.

### Drug solubility of FEL gel

The FEL gel dispersions (0.3 g of FEL gel was diluted with 10 mL of distilled water and stirred for 10 min) were centrifuged at 100,000 × *g* using Beckman Optima™ MAX-XP Ultracentrifuge (Beckman Coulter, Osaka, Japan) to separate the solubilized and non-solubilized FEL. The concentration of soluble FEL (solubility) was determined using HPLC as described above.

### Drug release from FEL gel

Drug release from FEL gel was measured according to our previous reports [17–19]. Briefly, reservoir chamber (12.2 mL) of Franz diffusion cell was filled with 0.2 mM phosphate buffer (pH 7.2), and membrane of pore size 20 μm was fixed on the cell. After that, 0.3 g of FEL gel (equivalent to 4.5 mg FEL) was spread uniformly over the membrane (*A*, 2 cm^2^) and incubated at 37 °C for 24 h. One hundred microliters aliquot samples were withdrawn from the reservoir chamber at 0.5, 1, 2, 3, 6, and 24 h. The FEL concentration and particle size distribution were measured using HPLC and NANOSIGHT, respectively, as described above. The area under the released FEL concentration-time curve (*AUC*_release_) was calculated using the trapezoidal rule up to the last measurement point (24 h).

### Skin penetration of FEL gel

Seventy-two Wistar rats (7-week-old) were divided into nine groups (8 rats/group), and the abdominal skin of the rats was shaved with an electric clipper and razor on the day prior to the experiment. After 24 h, the rats were euthanized by injecting a lethal dose (200 mg/kg) of pentobarbital. The abdominal skin was then removed and placed on a Franz diffusion cell. The in vitro skin penetration of FEL gel was investigated, as described in our previous study [17–19]. Briefly, the reservoir chamber (12.2 mL) was filled with 0.2 mM phosphate buffer (pH 7.2), and 0.3 g of FEL gel (equivalent to 4.5 mg FEL) was uniformly spread over the membrane (*A*, 2 cm^2^), and incubated at 37 °C for 24 h. Samples (100 µL) were withdrawn from the reservoir chamber at 0.5, 1, 2, 3, 6, and 24 h. The FEL concentration and particle size distribution were measured using HPLC and NANOSIGHT, respectively, as described above. In this study, endocytosis was inhibited by either low temperature incubation or using pharmacological inhibitors. Low temperature inhibition of endocytosis experiment was performed at 4 °C. Different pharmacological inhibitors such as 54 µM nystatin [caveolae-mediated endocytosis (CavME) inhibitor] [20], 40 µM dynasore [clathrin-mediated endocytosis (CME) inhibitor] [21], 2 µM rottlerin [micropinocytosis (MP) inhibitor] [22], and 10 µM cytochalasin D (phagocytosis inhibitor) [20] were used in experiments under thermoregulated (37 °C) conditions. These pharmacological inhibitors were dissolved in 0.5% dimethyl sulfoxide (vehicle) and pretreated for 1 h before the experiment. During the experiment, these inhibitors were added to the reservoir chamber (0.2 mM phosphate buffer). In this study, the area under the penetrated FEL concentration-time curve (*AUC*_penetration_) was calculated using the trapezoidal rule up to the last measurement point (24 h). Moreover, pharmacokinetic parameters [penetration rate, *J*_c_; skin/preparation partition coefficient, *K*_m_; skin penetration coefficient, *K*_p_; diffusion constant within the skin, *D*; lag time, *τ*; thickness of the skin, *δ* (0.071 cm); amount of FEL (*C*_C_) in the reservoir chamber at time *t*, *Q*_t_; effective area of the skin] were calculated according to Eqs. 1 and 2:

1 $${{\textit{J}}_{\textit{c}}} = \frac{\textit{{Q}}_{\rm{{t}}}}{{{\textit{A}} \cdot \left( {{\textit{t}} - \tau } \right)}} = \frac{{{{D}} \cdot {{\textit{K}}_{\rm{m}}} \cdot {{\textit{C}}_{\rm{c}}}}}{\delta } = {{\textit{K}}_{\rm{p}}} \cdot {{\rm{C}}_{\rm{c}}}$$

2 $${\textit{ D = }}\frac{{{\delta ^{\rm{2}}}}}{{{\rm{6}}\tau }}$$

### Percutaneous absorption of FEL gel

Twenty-four Wistar rats (7-week-old) were divided into three groups (8 rats/group), and a cannula filled with 30 µg/mL heparin (silicone tubing; i.d. 0.5 mm, o.d. 1.0 mm) was inserted into the right jugular vein of Wistar rats under isoflurane anesthesia on the day prior to the experiment. The abdominal skin of the rats was shaved with an electric clipper and razor. After 24 h, 0.3 g of FEL ointment and gel (equivalent to 4.5 mg FEL) was spread uniformly over the effective area (2 cm^2^) of the abdominal skin. Then, 200 µL of blood was collected from the right jugular vein through a cannulation tube at 0.5, 1, 2, 3, 6, and 24 h. The blood samples were centrifuged at 24,000 × *g* for 20 min at 4 °C, and the plasma obtained was used for the measurement of plasma FEL concentration using HPLC as described above. The area under the FEL concentration-time curve (*AUC*_plasma_) was calculated using the trapezoidal rule up to the last measurement point (24 h).

### Accumulation of FEL in skin tissue

Seventy-two Wistar rats (7-week-old) were divided into nine groups (8 rats/group). On the day prior to the experiment, the abdominal skin of the rats was shaved with an electric clipper and razor. After 24 h, 0.3 g of FEL ointment and gel (equivalent to 4.5 mg FEL) was spread uniformly over the effective area (2 cm^2^) of the abdominal skin. At 3, 6, and 24 h after treatment with FEL formulations (ointment and gel), the rats were euthanized by injecting a lethal dose (200 mg/kg) of pentobarbital, and the abdominal skin was removed. The FEL formulations were wiped off from the skin surface, and homogenized in methanol to extract FEL; the homogenates were centrifuged at 20,400 × g for 20 min at 4 °C. FEL concentrations in the supernatants were measured using HPLC as described above.

### Statistical analysis

The sample numbers (n) are shown in figure legends, and data are expressed as the mean ± standard error (S.E.) of the mean. Statistical analysis was performed using the JMP ver. 5.1 (SAS Institute). Student’s *t*-test and one-way analysis of variance (ANOVA) followed by Dunnett’s multiple comparison were used for analyses. Statistical significance was set at *P* < 0.05.

## Results

### Characteristics of gel-based l-menthol-containing FEL solid nanoparticles

**Fig. 1:**
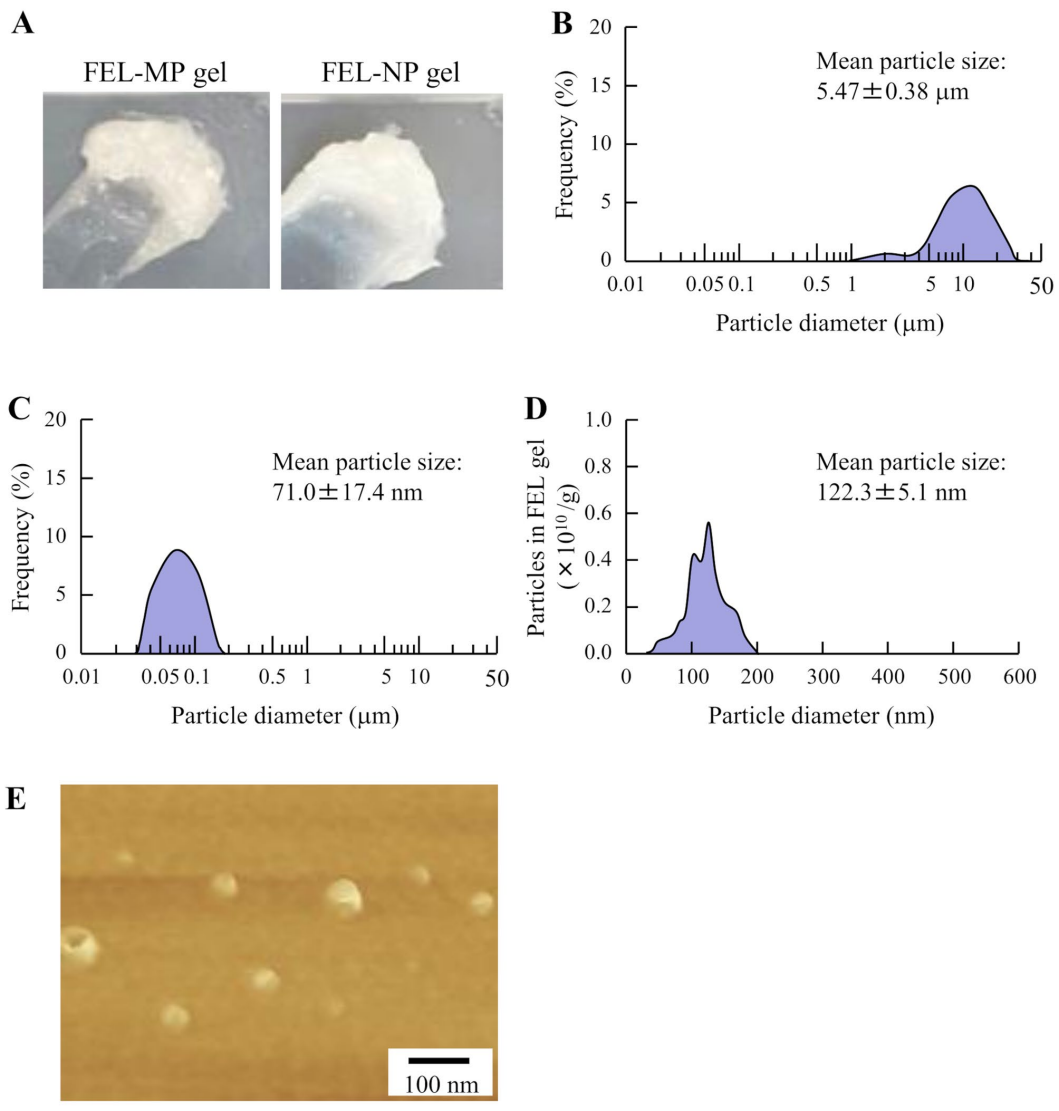
Digital image (**A**), particle size distribution (**B**-**D**) and AFM image (**E**) of FEL gels. The digital image (**A**) of FEL-MP and FEL-NP gels were monitored using digital camera, and the particle size distribution of FEL-MP (**B**) and FEL-NP (**C**) gels were measured by the SALD-7100. The particle size distribution of FEL-NP gel (**D**) was also detected by NANOSIGHT LM10. The AFM images of FEL-NP gels were obtained using SPM-9700. The FEL nanoparticles were detected in the gels, with particle size of 20–200 nm

**Fig. 2:**
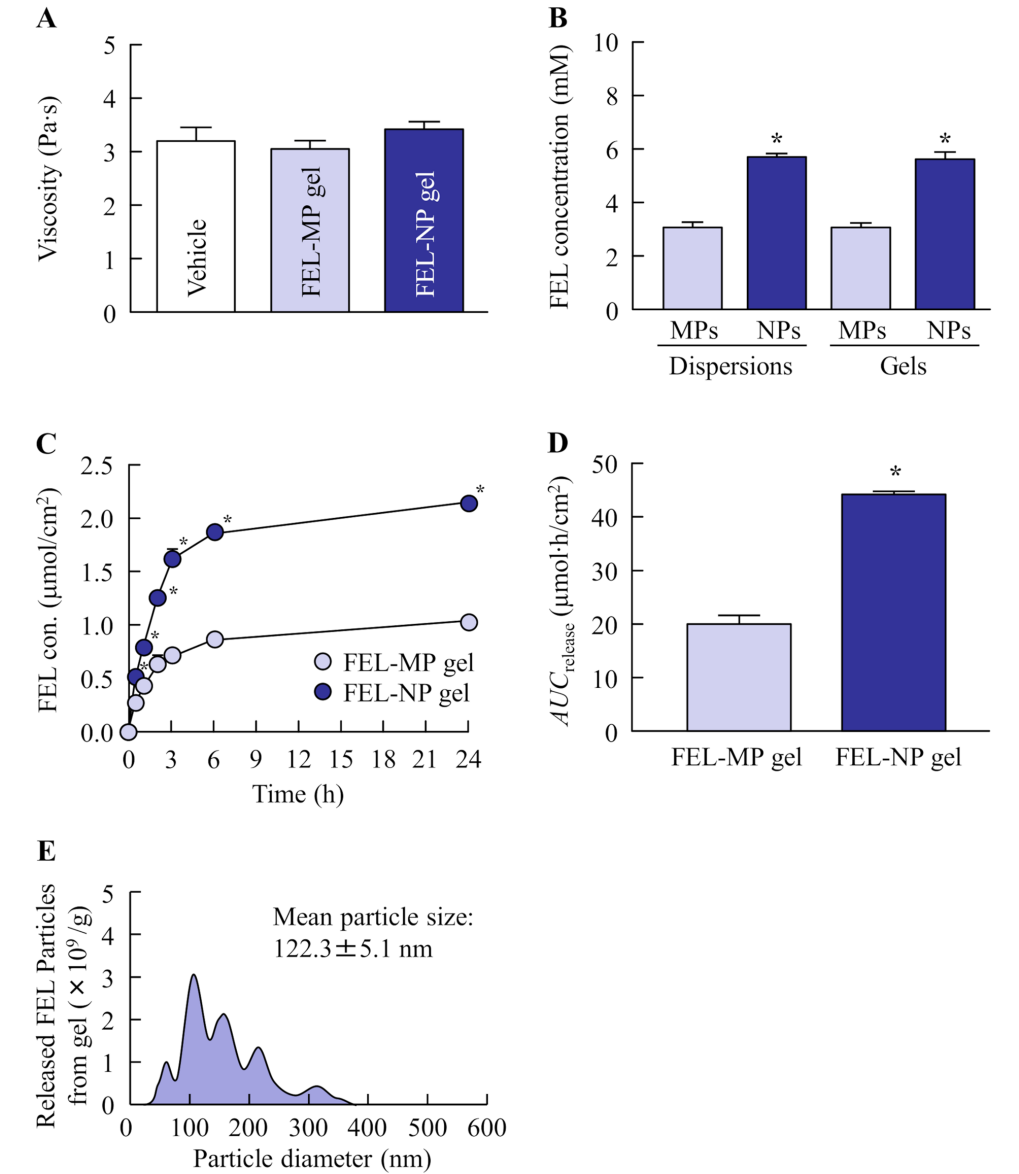
Viscosity (**A**), solubility (**B**), and drug release (**C**-**E**) of FEL-MP and FEL-NP gels. The solubility of FEL micro- and nanoparticles in FEL dispersions and gels was measured (**B**). A Franz diffusion cell was used to measure the release of FEL particles from the gel (**C**), and the particle size distribution of FEL released from the gel was measured using NANOSIGHT LM10 (**E**). n = 8. ^*^ *P* < 0.05, vs. FEL-MP gel for each category. Moreover, 92.1% FEL was present as solid particles, and FEL nanoparticles were released from the gel. The FEL concentration after release from FEL-NP gel was significantly higher than that from FEL-MP gel

Figure 1 shows the particle size of FEL in FEL-MP and FEL-NP gels. The particle size in FEL-NP gels subjected to bead milling was lower than that in FEL-MP gels, and the particle size was in the range of 20–200 nm. The FEL nanoparticles in the gel were spherical. Figure 2A and B show the viscosity and drug solubility of the gels, respectively. The viscosities of FEL-MP and FEL-NP gels were similar (Fig. 2A). The solubility of FEL was enhanced by bead mill treatment; however, gelation did not affect FEL solubility (Fig. 2B). The ratio of dissolved and solid FEL in FEL-MP and FEL-NP gels were 4.3:95.7 and 7.9:92.1, respectively. Figure 2 C-E show the drug release from FEL-MP and FEL-NP gels. The release of FEL from FEL-NP gels was significantly higher than that from FEL-MP gels, and the *AUC*_release_ of FEL-NP gels was 2.21-fold that of FEL-MP gels. Furthermore, both the dissolved FEL and FEL nanoparticles (size, 50–350 nm) were released from FEL-NP gels (Fig. 2E).

### Relationships of energy-dependent endocytosis and skin penetration of FEL gel

**Fig. 3:**
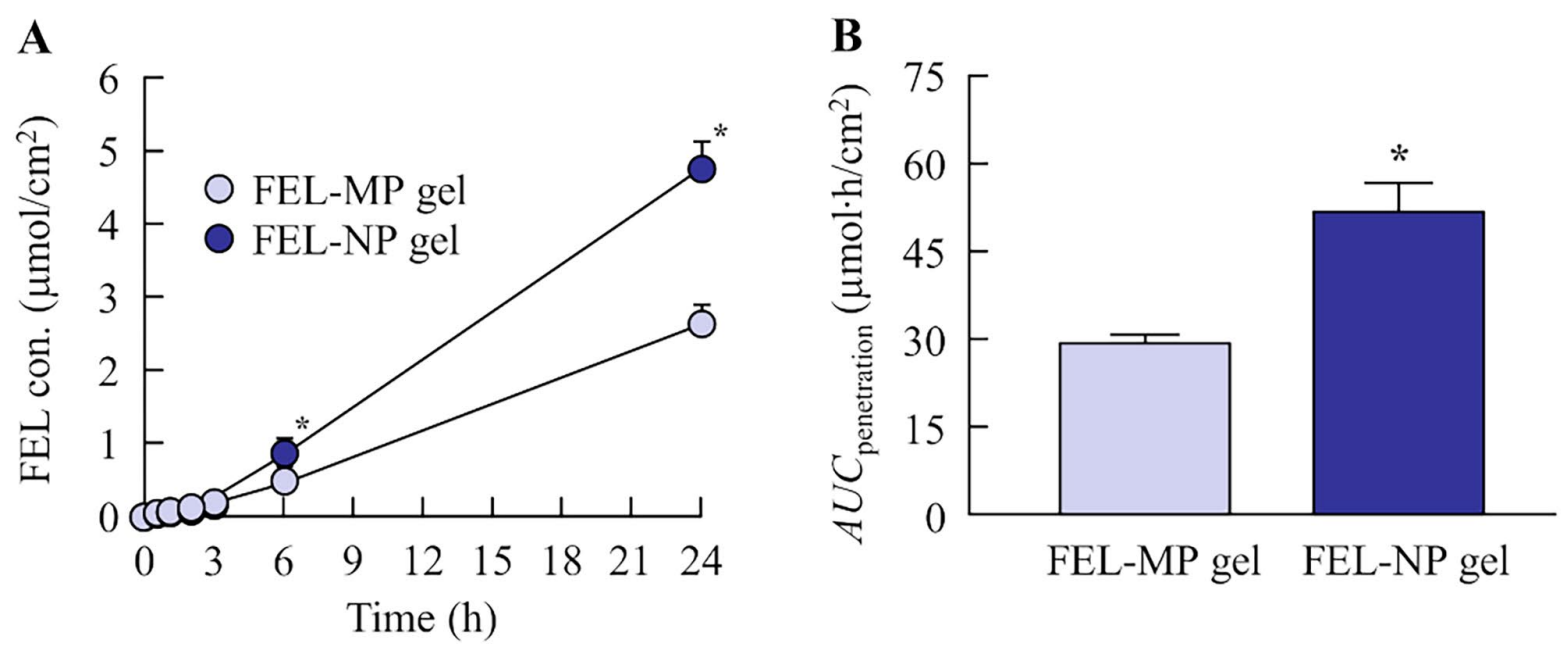
In vitro penetration profile (**A**) and *AUC*_penetration_ (**B**) of FEL-MP and FEL-NP gels. A Franz diffusion cell with rat skin was used. n = 8. ^*^ *P* < 0.05, vs. FEL-MP gel for each category. The penetration of FEL-NP gel was significantly increased compared with FEL-MP gel

**Fig. 4:**
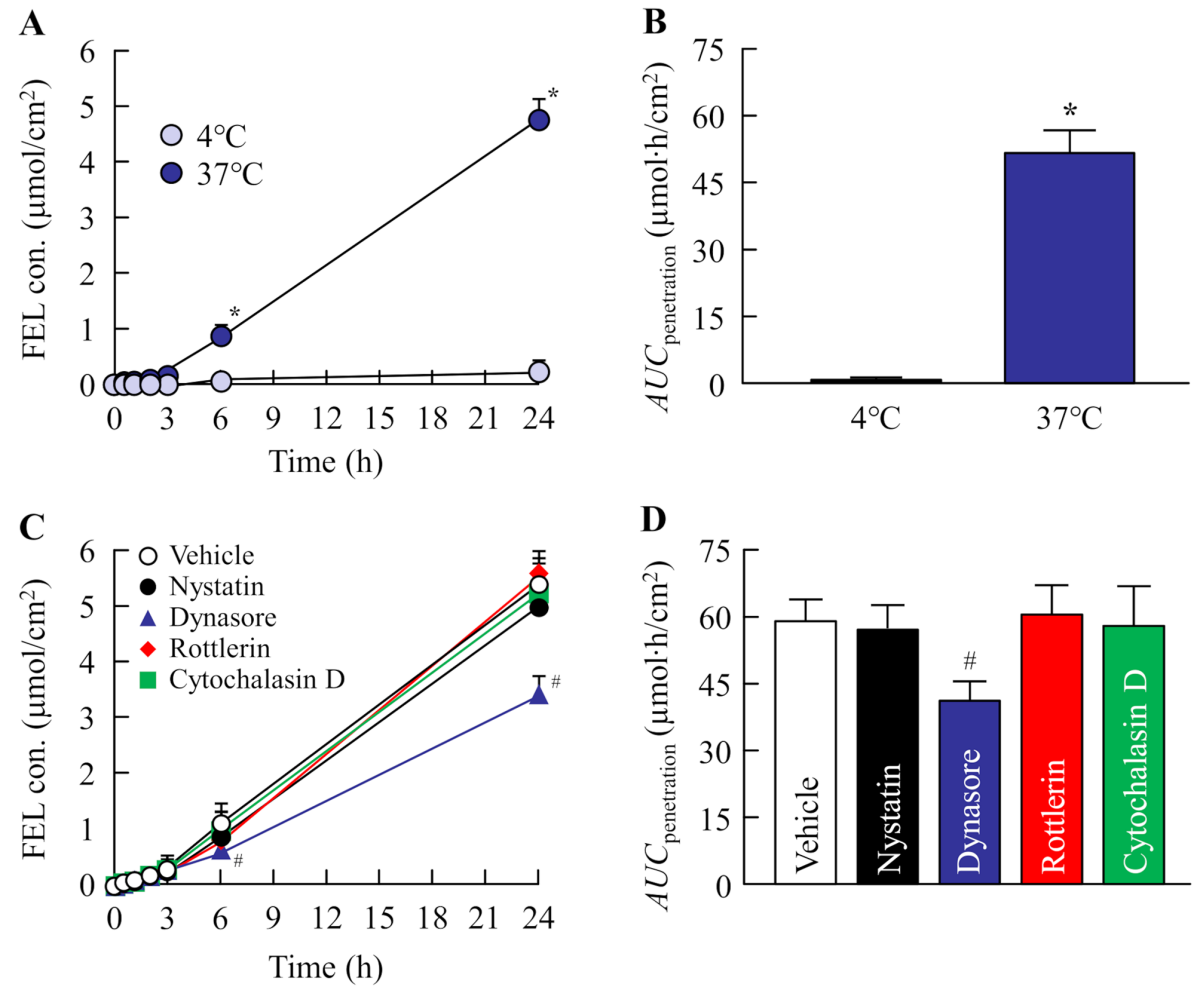
Relationships of energy-dependent endocytosis pathway and skin penetration of FEL in FEL-NP gel. A Franz diffusion cell with rat skin was used in the in vitro skin penetration experiments. Penetration profiles (**A**) and *AUC*_penetration_ values (**B**) of FEL-NP gel at 4 and 37 °C. Penetration profiles (**C**) and *AUC*_penetration_ values (**D**) of FEL-NP gel treated with endocytosis inhibitors (nystatin, dynasore, rottlerin, and cytochalasin D). n = 8. ^*^ *P* < 0.05, vs. 4 °C group for each category. ^#^*P* < 0.05, vs. vehicle for each category. The penetration of FEL-NP gel was significantly attenuated by inhibition of energy-dependent endocytosis, such as CME.

Figure 3 shows the penetration of FEL in rat skin treated with FEL-MP and FEL-NP gels, and Table 1 summarizes the pharmacokinetic parameters analyzed from the data in Fig. 3. The skin penetration of FEL from FEL-NP gels was significantly higher than that from FEL-MP gels, and the *AUC*_penetration_ of FEL-NP gels was 1.77-fold that of FEL-MP gels. Moreover, the *J*_c_, *K*_m_, and *K*_p_ of FEL-NP gel were higher than those of FEL-MP gel. In contrast with the result of drug release study (Fig. 2E), FEL nanoparticles were not detected in the reservoir chamber treated with FEL-NP gels. Previous studies have reported that the energy-dependent endocytosis pathway is related to the skin penetration of drug nanoparticles [17–19, 23]. Therefore, we investigated the effect of energy-dependent endocytosis on the skin penetration of FEL from FEL-NP gel (Fig. 4). The skin penetration of FEL from FEL-NP gel was strongly inhibited at 4 °C, an inhibitory condition for endocytosis (Fig. 4A and B). In addition, the skin penetration of FEL from FEL-NP gel was significantly attenuated by treatment with dynasore (a CME inhibitor).

**Table 1:** Pharmacokinetic analysis of in vitro penetration of FEL gel into rat skin

Gel	*J*_c_ (×10^− 2^ µmol/cm^2^/h)	*K*_p_ (×10^− 3^ cm/h)	*K*_m_ (×10^− 4^)	*τ* (h)	*D* (×10^− 3^ cm^2^/h)
FEL-MP gel	0.11 ± 0.004	0.03 ± 0.001	20 ± 0.03	0.96 ± 0.03	0.87 ± 0.03
FEL-NP gel	0.21 ± 0.015*	0.06 ± 0.004^*^	53 ± 0.39^*^	1.08 ± 0.15	0.77 ± 0.14

n = 8. ^*^ *P* < 0.05, vs. FEL-MP gel for each category

### Drug absorption behavior through the rat skin from FEL gel

**Fig. 5:**
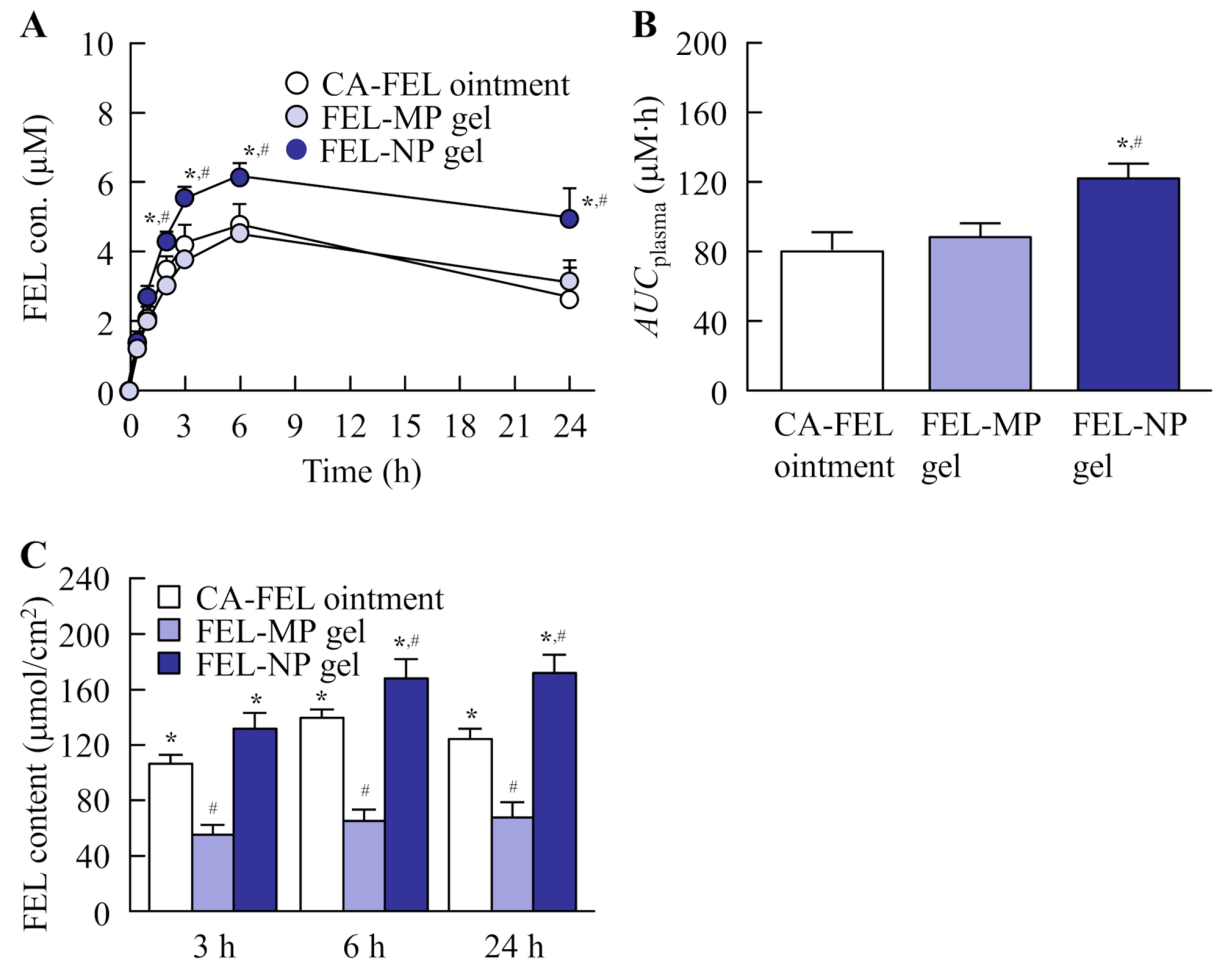
Percutaneous absorption profiles (**A**), *AUC*_plasma_ (**B**), and FEL amount in the skin (**C**) treated with FEL ointment and gel. The percutaneous absorption and drug amount in the skin were measured in 7-weeks-old Wister rats. n = 8. ^*^ *P* < 0.05, vs. FEL-MP gel for each category. ^#^*P* < 0.05, vs. CA-FEL ointment for each category. The percutaneous absorption and drug content in the skin treated with FEL-NP gels were significantly enhanced. The *AUC*_plasma_ of FEL-NP gels was 1.52- and 1.38-fold that of CA-FEL ointment and FEL-MP gel, respectively. Moreover, the FEL content in the rat skin treated with FEL-NP gels was 1.38- and 2.54-fold higher than that of CA-FEL ointment and FEL-MP gels, respectively, after 24 h of treatment

Figure 5 A and 5B show the percutaneous absorption of FEL-MP and FEL-NP gels. FEL was detected in the blood of rats treated with FEL gels for 24 h, and the percutaneous absorption in rats treated with FEL-NP gel was significantly higher than that in rats treated with FEL-MP gel. Figure 5 C shows the FEL concentration in the skin tissue of rats treated with FEL-MP and FEL-NP gels. The FEL concentration in the skin was also significantly higher for FEL-NP gel compared with FEL-MP gel, and the FEL concentration in the skin of rats treated with FEL-NP gel was 2.54-fold that of FEL-MP gel at 24 h after application of FEL gel. In addition, the FEL concentration in the blood and skin of rats treated with FEL-NP gel was significantly higher than that in rats treated with CA-FEL ointment.

## Discussion

It is important to design an effective formulation to enhance the skin penetration of FEL. In this study, we successfully prepared formulation (FEL-NP gel) containing *l*-menthol and FEL solid nanoparticles for topical application, and showed high drug release and local and systemic absorption after application of FEL-NP gel compared with FEL-MP gel. In addition, we found that high skin permeability from FEL-NP gel was attributed to the CME pathway.

In the breakdown method used for producing drug nanoparticles, additives are important for effective milling and dispersibility. Our previous study showed that MC enhanced the crushing efficiency, and HPβCD prevented the aggregation of drug particles during bead mill treatment. Based on these findings, we prepared dispersions containing FEL nanoparticles using these additives (MC and HPβCD), and successfully achieved FEL nanoparticles of size 20–120 nm. Next, we attempted to prepare topical formulations (dermal and transdermal) containing FEL nanoparticles. The selection of an appropriate formulation base is essential for the ease of application and improved dermal and transdermal delivery. A high moisture content is favorable for reducing skin irritation and keeping the skin hydrated, and a base prepared using minimal organic solvents and having excellent air permeability, minimal dermal irritation, good biocompatibility with skin, and high drug loading capacity is a prerequisite in skin formulations. Hydrogels, such as carboxypolymethylene, are favored in biomedical applications and used in broad range of pharmaceuticals; they have three-dimensional, hydrophilic, polymeric networks capable of imbibing large amounts of biological fluids and water [24–26]. Our previous studies reported that nanoparticles incorporated in carboxypolymethylene gel bases showed high drug release from the formulation [17–19, 27]. Moreover, we observed that *l*-menthol enhanced the penetration of drug nanoparticles by altering the barrier properties of the SC. Hence, in this study, we selected carboxypolymethylene gel bases to incorporate FEL nanoparticles and added *l*-menthol as skin permeation enhancer. The FEL particle size in FEL-NP gel was 20–200 nm (Fig. 1), and 92.1% of FEL in FEL-NP gel was solid nanoparticles (Fig. 2B). Furthermore, we demonstrated drug release from the gel (Fig. 2). The release of FEL from FEL-NP gels was significantly higher than that from FEL-MP gels, and the FEL nanoparticles was observed in the released FEL from the gel (Fig. 2E). These results confirmed the quality of FEL-NP gel as a dermal and transdermal formulation incorporating FEL nanoparticles.

Next, we demonstrated whether the skin penetration of FEL was enhanced through rat skin treated with FEL-NP gel (Fig. 3). Viscosity was similar for both FEL-MP and FEL-NP gels (Fig. 2A); however, skin/preparation partition coefficient (*K*_m_) and skin penetration coefficient (*K*_p_) of FEL-NP gels were high, and the penetration rate (*J*_c_) of FEL in FEL-NP gels was 1.91-fold higher than that of FEL-MP gel. It is important to demonstrate the mechanism of high skin penetration of FEL nanoparticles. A recent study reported that nanoparticles permeate deep into the skin depending on their surface charge and size [28]. Moreover, *l*-menthol spreads through the gap in the SC cells and decreases its barrier properties, leading to skin penetration of drug nanoparticles through the barrier [29]. Our previous study using raloxifene also showed that the skin penetration of over 100 nm particles was a little, however, addition of *l*-menthol enhanced the penetration of drug nanoparticles by altering the barrier function of the SC, resulting in allowing drug particles that were 100–450 nm in size to transit into the underlying epidermis, resulting in enhancement of the local and systemic absorption [17–19]. The FEL particle size in FEL-NP gel in this study was less than 200 nm (Fig. 1). Therefore, both the particle size and use of *l*-menthol as skin permeation enhancer may be related to the passage of the drug through the SC. In addition, it has been reported that the energy-dependent endocytosis pathway promotes the uptake of drug nanoparticles in the epidermal layer of the skin [17–19]. In this study, the skin penetration from FEL-NP gel was low at 4 °C, a temperature that inhibits all energy-dependent endocytosis (Fig. 4A and B). Moreover, the CME inhibitor dynasore attenuated the skin penetration of FEL in FEL-NP gel (Fig. 4C and D). These results suggest that skin penetration of FEL nanoparticles is accelerated by the CME pathway. Moreover, FEL nanoparticles were dissolved during the penetration process, as FEL nanoparticles was not detected in the reservoir chamber treated with FEL-NP gels (Fig. 3). Further, FEL skin penetration from FEL-NP gel at 4 °C was remarkably lower than that from FEL-NP gel treated with dynasore (Fig. 4). At a temperature of 4 °C, not only endocytosis dysfunction occurs but the gap in the SC cells is also narrowed, which may be related to the difference in skin penetration of FEL-NP gel. However, further studies are required to clarify these mechanisms.

In general, it is possible to compare the drug release from the gel and skin penetration in the in vitro experiments, however the in vitro experiment is not able to reflect the correct in vivo profile, since the properties in the reservoir side affect the release profile of the drug from the gel in the in vitro study. For this reason, the in vivo studies using laboratory animals are need to evaluate the skin penetration of gel formulation. In this study, we also investigated the local and systemic absorption of FEL-NP gel in the in vivo study using rats (Fig. 5). Although, the local absorption of FEL-MP gel was lower than that in that of CA-FEL ointment containing 3% FEL, the FEL-MP gel showed similar *AUC*_plasma_ with the CA-FEL ointment. It was known that the high lipophilicity enhance drug permeation through the SC, however, the excessive lipophilicity attenuate the passive diffusion of drug to dermis from the skin epidermis, since the skin dermis is more hydrophilic than the skin epidermis. These lipophilicity in the formulations may relate the difference in systemic and local absorption between FEL-MP gel and CA-FEL ointment. On the other hand, both local and systemic absorption of FEL were significantly increased in FEL-NP gel, and high FEL concentration in the skin tissue and blood were observed for 1 day (24 h). In addition, local and systemic absorption of FEL in FEL-NP gel (1.5% FEL) was significantly higher than that of CA-FEL ointment containing 3% FEL. These results showed that topical formulations based on FEL nanoparticles, *l*-menthol, and hydrogels were useful as both dermal and transdermal formulations, and nanoparticle-based gel formulations may provide equivalent or better drug efficacy at about half drug dosage in CA-FEL ointment. Thus, we found that skin formulations based on FEL nanoparticles (FEL-NP gel) provided high local tissue concentration and systemic absorption of FEL in this study. These findings are important to design the new transdermal therapeutic systems.

In this study, the systemic absorption of FEL in FEL-NP gel were significantly higher in comparison with CA-FEL ointment, and the enhanced plasma FEL may cause to the disadvantage of increased systemic side effects. Therefore, further studies, which of changing in the drug concentration and the viscosity in the ointment, need for the practical application of FEL nanoformulations. Moreover, it is important the measuring of the in vitro information on CA-FEL ointment to elucidate the usefulness of FEL-NP gel. In addition, we are investigating in a future study whether topical application of FEL-NP gel attenuates inflammation of adjuvant-induced arthritis in rats.

## Conclusion

We prepared skin formulations based on FEL nanoparticles, *l*-menthol, and hydrogels (FEL-NP gel). The FEL-NP gel provided high local tissue concentration and systemic absorption of FEL. The drug penetration route is as follows: release from the formulation (I), diffusion into the SC (II), epidermal layers (III), dermis (IV), and absorption by the capillary network (V) [30]. Taken together, we hypothesized that the penetrated FEL nanoparticles were taken up into cells (viable epidermis) by energy-dependent endocytosis. During this process, FEL diffused into the epidermis, dermis, and subcutaneous tissue layers, resulting in enhanced local and systemic absorption. These findings provide useful information for the design of topically applied nanoformulations against inflammation by providing local and systemic effects.

## Electronic supplementary material

Below is the link to the electronic supplementary material.

Supplementary Material 1

Supplementary Material 2

## Data Availability

The data generated in the present study may be requested from the corresponding author.

## Abbreviations

AFM: Atomic force microscope
*AUC*: Area under the indomethacin concentration-time curve
CA-FEL ointment: Commercially available FEL stick ointment
CavME: Caveolae-mediated endocytosis
CME: Clathrin-mediated endocytosis
*D*: Diffusion constant within the skin
FEL: Felbinac
FEL-NP gel: Carboxypolymethylene gel based FEL nanoparticles and *l*-menthol
FEL-MP gel: Carboxypolymethylene gel based FEL microparticles and *l*-menthol
HPβCD: 2-hydroxypropyl-β-cyclodextrin
*J*_c_: Penetration rate
*K*_m_: Skin/preparation partition coefficient
*K*_p_: Penetration coefficient through the skin
MC: Methylcellulose
MP: Micropinocytosis
NSAIDs: Non-steroidal anti-inflammatory drug
SC: Stratum corneum
S.E.: Standard error
*τ*: Lag time
