# Supplementary material for: Skin absorption of felbinac solid nanoparticles in gel formulation containing l-menthol and carboxypolymethylene
Source: J Pharm Health Care Sci. 2023 Jun 6;9:20. doi: 10.1186/s40780-023-00290-1 (PMC10243057; doi:10.1186/s40780-023-00290-1)
Supplement: Supplementary file 2 — Supplementary Material 2 [file 40780_2023_290_MOESM2_ESM.pdf]

editage

Editing Certificate

This document certifies that the manuscript listed below has been edited to ensure language and grammar accuracy and is error free in these aspects. The logical presentation of ideas and the structure of the paper were also checked during the editing process. The edit was performed by professional editors at Editage, a division of Cactus Communications. The sections titled references, figures, references and figures were not edited by Editage upon the author's request.

The author's core research ideas were not altered during the editing process. Editage guarantees the quality of editing with the assumption that our suggested changes have been accepted and the edited text has not been altered without the knowledge of our editors.

MANUSCRIPT TITLE

Usefulness of felbinac solid nanoparticles and l-menthol in carboxypolymethylene gel based dermal and transdermal formulations

AUTHORS

Reita Kadowaki Fumihiko Ogata Aoi Fushiki Saki Daimyo Saori Deguchi Hiroko Otake Mayumi Nagata Hiroshi Sasaki Naohito Kawasaki1, Noriaki Nagai

ISSUED ON

January 23, 2023

JOB CODE

EBWRU_8

THE EDITAGE Promise QUALITY GUARANTEE

Vikas Narang

Vikas Narang
Chief Operating Officer - Editage

editage

Editage, a brand of Cactus Communications, offers professional English language editing and publication support services to authors engaged in over 1300 areas of research. Through its community of experienced editors, which includes doctors, engineers, published scientists, and researchers with peer review experience, Editage has successfully helped authors get published in internationally reputed journals. Authors who work with Editage are guaranteed excellent language quality and timely delivery.

GLOBAL : +1(833) 979-0061 | request@editage.com

JAPAN : 0120-50-2987 | submissions@editage.com

CACTUS

IMPACT SCIENCE impact.science

researcher.life

CACTUS LIFE SCIENCES lifesciences.cactusglobal.com
