# Supplementary material for: Skin absorption of felbinac solid nanoparticles in gel formulation containing l-menthol and carboxypolymethylene
Source: J Pharm Health Care Sci. 2023 Jun 6;9:20. doi: 10.1186/s40780-023-00290-1 (PMC10243057; doi:10.1186/s40780-023-00290-1)
Supplement: Supplementary file 1 — Supplementary Material 1 [file 40780_2023_290_MOESM1_ESM.docx]

May 1, 2023

Journal of Pharmaceutical Health Care and Sciences

Editor Prof. Yukinobu Kodama

Manuscript ID. JPHC-D-23-00010R2: “**Skin absorption of felbinac solid nanoparticles in gel formulation containing *l*-menthol and carboxypolymethylene**”

Dear Prof. Dr. Yukinobu Kodama:

Thank you for your kind letter of May 1, 2023. As you and the reviewers suggested, I have revised our manuscript and attach a list of responses to the reviewers’ comments. I am sending this list here with our revised manuscript.

I hope that the revised manuscript will be accepted for publication in **Journal of Pharmaceutical Health Care and Sciences.**

Sincerely yours,

Noriaki Nagai, Ph.D.

Faculty of Pharmacy, Kindai University

Tel : +81-6-4307-3638

Fax : +81-6-6730-1394

E-mail : nagai_n@phar.kindai.ac.jp

**Response to Reviewer Comments**

We carefully revised our manuscript according to the suggestions of the reviewers, and details are as follows.

**< Q and A for Reviewer 1>**

**Q5-1. The FEL concentration of the prepared gel formulation was 1.5%, which is different from the CA-FEL 3% formulation. Please clarify the reason for the setting formulation.**

A5-1. The reviewer’s comment is correct. The plasma FEL levels and FEL content in the rat skin treated with FEL-NP gels were increased by using solid nanoparticles, and the enhanced the local and systemic absorption of FEL in the FEL-NP gel (1.5%) was better absorption in comparison with CA-FEL ointment containing twice the content (3%). The result suggest that nanoparticle-based gel formulations may provide equivalent or better drug efficacy at about half drug dosage in CA-FEL ointment. In this study, we selected the 3% CA-FEL ointment to perform this comparison. In order to respond to the reviewer’s comment, we added these descriptions in the Discussion. Thank you very much for pointing this out (line 383-388).

**Q5-2. As in the authors' response, it is well understand that the NP gel enhances the skin absorption. However, 1.5% MP-gel, which in microparticle as well as CA ointment, showed similar AUC plasma with CA ointment. On the other hand, intradermal FEL content of MP-gel is lower than that of CA ointment. The difference in systemic and local absorption between MP-gel and CA ointment should be discussed.**

A5-2. The reviewer’s comments are very important. The high lipophilicity enhance drug permeation through the stratum corneum, however, the excessive lipophilicity attenuate the passive diffusion of drug to dermis from the skin epidermis, since the skin dermis is more hydrophilic than the skin epidermis. These lipophilicity in the formulations may relate the difference in systemic and local absorption between MP-gel and CA ointment. In order to respond to the reviewer’s comment, we added these descriptions in the Discussion. Thank you very much for pointing this out (line 376-383, 394-395).

Thank you for great comments.
